# Reproductive Characteristics of a *Populus euphratica* Population and Prospects for Its Restoration in China

**DOI:** 10.1371/journal.pone.0039121

**Published:** 2012-07-26

**Authors:** Dechang Cao, Jingwen Li, Zhenying Huang, Carol C. Baskin, Jerry M. Baskin, Peng Hao, Weilei Zhou, Junqing Li

**Affiliations:** 1 Key Laboratory for Silviculture and Conservation of Ministry of Education, Beijing Forestry University, Beijing, China; 2 State Key Laboratory of Vegetation and Environmental Change, Institute of Botany, Chinese Academy of Sciences, Beijing, China; 3 Graduate University of Chinese Academy of Sciences, Beijing, China; 4 Department of Biology, University of Kentucky, Lexington, Kentucky, United States of America; 5 Department of Plant and Soil Sciences, University of Kentucky, Lexington, Kentucky, United States of America; USDA-ARS, United States of America

## Abstract

*Populus euphratica* is a dominant tree in riparian ecosystems in arid areas of northwest China, but it fails to regenerate in these systems. This study evaluates causes for the failure of sexual and asexual regeneration of this species in the wild. *P. euphratica* disperses as many as 85743 seeds/m^2^ during summer, and the seeds germinate to 92.0% in distilled water and to 60.8% on silt. However, very few seeds (3.6%) can germinate on unflooded soil. The seed-rain season is prolonged by temporal variability in seed dispersal among individuals, which ensures that seedling emergence can occur during favorable conditions (i.e., floods and rainfall). As a result of water shortage and river channeling due to water usage and altered river flows, there are no safe sites on river banks for seed germination, which has led to the failure of *P. euphratica* to regenerate from seed. Root suckers of *P. euphratica* were present in 86% of the forest gaps investigated. However, extensive grazing has destroyed many of them and thus has reduced this form of regeneration. This research suggests that human activities are resulting in the failure of *P. euphratica* to regenerate. Changes in land management such as reduced use of concrete canals in *Populus* forests and/or reduced sheep grazing in these areas may promote their regeneration.

## Introduction

Plant adaptation and reproductive strategies occur over a long evolutionary time, while present human disturbances alter the environment rapidly. Such maladjustment leads to severe endangerment and even local extinction of some species [1], [2]. Cottonwoods (*Populus* spp.) are severely affected by human disturbances to riparian ecosystems in many parts of the world [3], [4], [5], [6]. Human activities not only affect cottonwood recruitment directly via cattle grazing, but they also change the environments of riparian habitats [7]. River regulation (damming and dewatering) via changing hydrological regimes is an important factor contributing to degradation of riparian cottonwood forests [4], [8]. In addition, extirpation of mammalian predators has also been reported to be a factor affecting recruitment of cottonwoods via increasing browsing pressure of wild ungulates on seedlings and saplings [9].

*Populus euphratica* is a dominant tree species of the riparian forests in arid areas of Central Asia [10], [11], [12], [13], where it plays an important role in stabilizing these vulnerable ecosystems [14]. However, *P. euphratica* forests have been in rapid decline due to water shortage and water pollution [15], [16], [17]. Previous studies indicated that groundwater levels and flooding regimes played important roles in regeneration and development of *P. euphratica* populations [6], [18], [19], [20], [21]. Various efforts have been made in China for land recovery from the severe environmental degradation, including several national projects to allocate some water to areas of *P. euphratica* [22]. However, there is still a continual degradation of *P. euphratica* forests [23].

*P. euphratica* has the ability to regenerate by both seed/seedlings and root suckers [12]. To understand the decline of *P. euphratica*, attention should be paid to both seedling establishment and sucker establishment. In field investigations, we observed two distinct kinds of forest gaps in which *P. euphratica* produces root suckers. In some forest gaps with >50% canopy covering, there were very high densities of root suckers and mortality was very low, while in other gaps, there were many fewer root suckers, and mortality was high. We also found that adult individuals in a stand were usually of similar ages. The purpose of the present study was to (1) examine regeneration strategies of *P. euphratica* by seed/seedlings and root suckers, (2) test if *P. euphratica* has some problems in its reproduction and (3) to clarify any constraints found on its reproduction and define how they function. Thus, we investigated seed dispersal and germination of *P. euphratica* and its adaptations to flooding regimes. We also investigated establishment of root suckers in the field and the effect of sheep grazing on sucker establishment. On the basis of these experiments and investigations, we discussed the effects of human activities on regeneration of *P. euphratica* forests.

## Results

### Seed dispersal of *P. euphratica*

**Figure 1:**
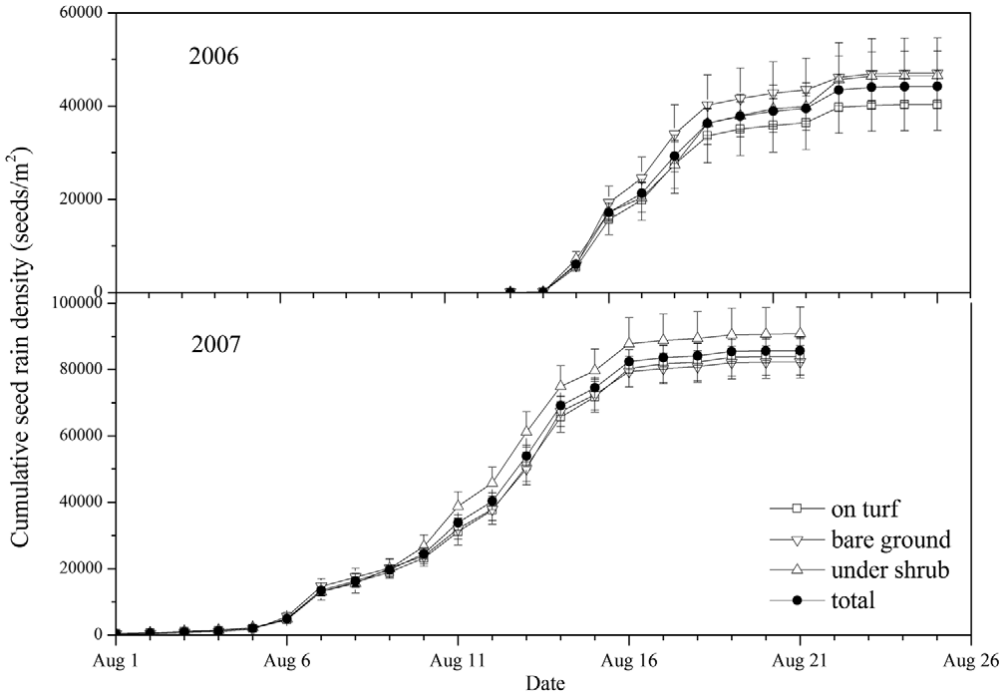
Cumulative seed rain density in plot 1 in 2006 and 2007.

*P. euphratica* disperses large numbers of seeds during seed rain seasons. The cumulative seed rain density (hereafter SRD) reached 44204±3406 (mean ± s.e., with 95% probability of confidence interval) and 85743±3617 seeds per m^2^ in 2006 and 2007, respectively (Fig. 1). There was no significant influence of microhabitat on cumulative SRD (p>0.05). Duration of the seed rain season at plot 1 was 14d in 2006 and 21d in 2007.

The duration of seed dispersal for individual trees was 5.6 to 9.4 days, and seed dispersal began on different dates (Table 1). In 2008, the earliest starting date of seed dispersal was 14 July and the latest was 28 August. There was a 45-d duration of seed dispersal for the *P. euphratica* population.

**Table 1:** Temporal variability of *P. euphratica* seed dispersal among individuals.

Plot No.	Duration (d)			Starting date		Ending date	
	Mean	Min	Max	Earliest	Latest	Earliest	Latest
1	8.7	2	28	18 Jul.	14 Aug.	7 Aug.	15 Aug.
2	9.4	3	35	14 Jul.	25 Aug.	19 Jul.	27 Aug.
3	5.6	2	30	18 Jul.	28 Aug.	24 Jul.	After 28 Aug *

* One individual started seed dispersal on 28 August, 2008 when the observation was finished, thus the ending date of its seed dispersal was recorded as “after 28 Aug”.

### Germination of *P. euphratica* seeds on different substrates

**Figure 2:**
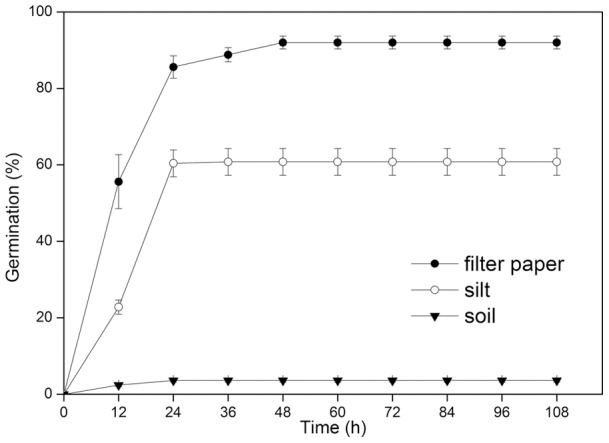
Germination percentage of *P. euphratica* seeds incubated on filter paper, soil and silt.

Freshly-dispersed *P. euphratica* seeds germinated to 92.0±1.7% (mean ± s.e.) on filter paper, to 60.8±3.5% on river silt, and to only 3.6±0.4% on forest soil (p<0.001, Fig. 2).

### Establishment of *P. euphratica* root suckers and environmental factors

Root suckers occurred in 86% of all forest gaps investigated; however, root suckers died out in 24% of the gaps. Both height and basal diameter of root suckers were positively associated with density of *Sophora alopecuroides* (p<0.01). Height of root suckers was positively associated with mean distance to nearest adult trees (p<0.05) and negatively associated with sheep grazing (p<0.01). In addition, there was a positive association between basal diameter of root suckers and number of adult *P. euphratica* individuals (p<0.05, Table 2). However, there was no significant correlation between densities of root suckers and either environmental factors or other plants present in the forest (Table 2).

**Table 2:** Correlation between influencing factors and growth characteristics of *P. euphratica* root suckers.

Influencing factors	Density	Height	Basal diameter
Density of *Sophora alopecuroides*	−0.16	0.250**	0.25**
Soil moisture at depth of root suckers	−0.078	0.078	0.126
Degree of soil compactness	−0.19	−0.097	0.018
Adult individuals of *P. euphratica*	−0.244	0.094	0.163*
Mean distance to nearest trees	0.024	0.170*	0.109
Sheep grazing	0.053	−0.370**	−0.048

Significance: **P<0.01; *P<0.05.

### Regeneration of *P. euphratica* root suckers in different kinds of forest gaps

**Figure 3:**
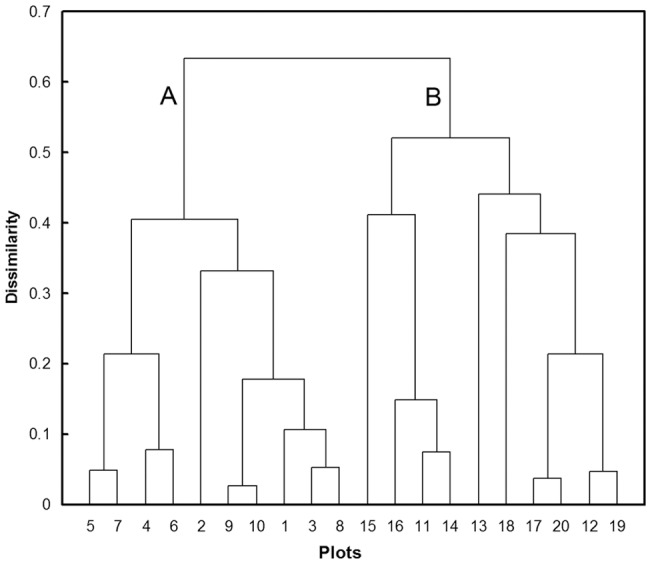
Cluster analysis dendrogram of the 20 plots investigated in the third observation.

Hierarchical Cluster Analysis (HCA) showed that the 20 forest gaps clustered into two groups (Fig. 3). Group A had greater density (p<0.05) and lower mortality (p<0.01) of suckers than group B (Table 3). Forest gaps of group A had an average density of 196.4±71.1 root suckers per 100 m^2^ and an average mortality of 0.5±0.2%. Group B had an average density of 4.2±1.2 root suckers per 100 m^2^ and an average mortality of 60.3±6.7%. There was no significant difference between the two groups of gaps in height, basal diameter or soil depth where root suckers sprouted (Table 3). Root suckers sprouted on lateral roots with an average diameter of 0.87∼0.93 cm, at a mean soil depth of 14.7∼24.8 cm.

**Table 3:** Growth characteristics of two groups of *P. euphratica* root suckers in forest gaps.

Forest gaps	Density (100m^−2^)*	Mortality (%)**	Height (cm)	Basal D (cm)	Depth (cm)	Root D (cm)
Group A	196.4±71.1	0.5±0.2	73.3±6.7	0.69±0.04	14.7±1.3	0.87±0.03
Group B	4.2±1.2	60.3±6.7	72.2±8.9	0.65±0.07	24.8±4.9	0.93±0.09

Values are means ± s.e. Basal D, basal diameter of root suckers; Root D, diameter of roots on which root suckers sprouted. Significance: **P<0.01; *P<0.05.

Standard deviations of ages of *P. euphratica* individuals in a stand were 0.3 to 3.7 yr (Table 4), and coefficient of variation (CV) of individual ages ranged from 6% to 25%. It was obvious that *P. euphratica* individuals in a given stand were of similar age.

**Table 4:** Age structure of *P. euphratica* at the sites investigated in the fourth observation.

Plot No.	Plot size (m^2^)	Density (100 m^−2^)	Mean age (yr)	SD	CV (%)
1	467.5	38	18.1	3.6	20.02
2	568.3	132	5.1	0.3	6.41
3	1490.6	8	11.9	2.2	18.29
4	1866.9	52	15.1	3.7	24.53
5	1658.6	48	12.9	3.2	24.89
6	412.4	36	14.6	2.0	14.02
7	274.6	40	18.4	3.5	19.23
8	1076.1	28	16.1	2.7	16.52
9	592.0	44	17.2	3.2	18.53
10	358.3	32	16.7	2.9	17.36
11	398.5	52	16.6	2.9	17.53
12	413.6	32	16.2	2.8	17.59
13	304.4	32	17.4	2.8	16.27
14	711.6	44	16.9	2.3	13.89
15	531.5	24	17.8	3.4	18.88
16	287.2	48	14.1	3.0	21.34
17	146.6	60	11.4	1.7	15.36
18	508.4	28	19.2	2.6	13.31
19	1489.6	32	15.7	2.0	12.68
20	798.3	32	16.9	3.1	18.34

SD is standard deviation, and CV is coefficient of variation.

### Effect of soil moisture on occurrence of root suckers

**Figure 4:**
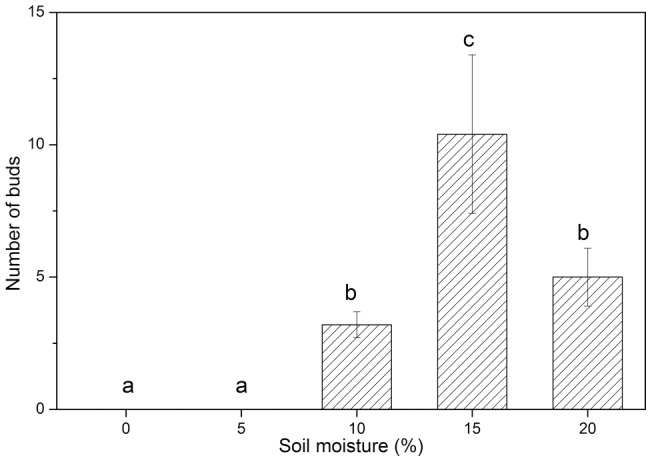
Number of buds on root sections buried in soil at different moisture contents. Values with the same letter are not significantly different (p>0.05).

The occurrence of root suckers was positively correlated with soil moisture (p<0.0001, Fig. 4). No buds occurred in soil with less than 5% moisture, and the amount of buds increased with an increase of soil moisture (Fig. 4). The highest number of buds was 10.4±3.0 in soil moisture of 15%. However, the number of buds decreased with a further increase (>15%) of soil moisture (Fig. 4).

**Figure 5:**
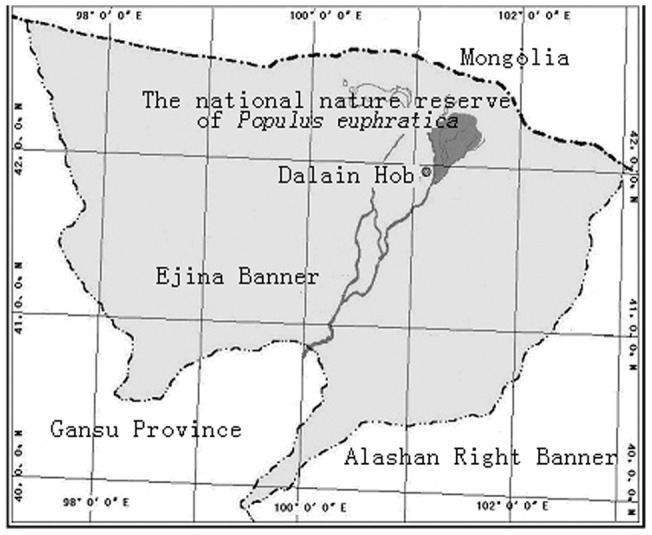
Location of study area. All field investigations were conducted in the National Natural Reserve of *Populus euphratica* in Ejina Oasis, NW China (41°30′∼42°07′N, 101°03′∼101°17′E).

## Discussion

*Populus euphratica* produced and dispersed large numbers of seeds during the seed-rain-season of every year observations were made. Seed-rain duration of the whole population is prolonged by asynchrony in seed dispersal among individuals, which increases the possibility for seeds to experience flooding and rainfall events. Although seed dispersal of individuals was asynchronous, dispersal of most seeds of *Populus euphratica* is synchronized with the occurrence of the highest runoff and precipitation in NW China [24]. Thus, the seed dispersal strategy fits the hydrologic dynamics in this area, and the seeds could germinate on wet floodplains. As a result, *P. euphratica* is distributed widely along riverbanks [15].

Most *P. euphratica* seeds are viable, and they germinate better on flooded river banks than in nonflooded forests. Salinity and plant allelochemicals in soils of *P. euphratica* forests inhibit seed germination of *P. euphratica* [5]. Therefore, seedling recruitment of *P. euphratica* can only occur on floodplains leached by floods and with adequate soil moisture. However, there has been an increasing overuse of water in the upper and middle reaches of the Heihe River, and many dams and concrete irrigation canals have been built within the distribution areas of *P. euphratica* to ensure a water supply for agricultural use [12]. As a result, there are no humid habitats on river banks for seed germination. Removal of concrete canals may restore the natural flooding regime of these riparian areas and provide opportunities for natural regeneration of *P. euphratica* forests. Thousands of seedlings emerge on alluvial deposits on river banks every year in July and August when floods recede, but they soon die of drought. For another cottonwood (*P. nigra* L.), no seedlings survive if the change from wet to dry conditions lasts for more than one week [25]. It also seems true for *P. euphratica* that long durations of wet substrate help ensure seedling survival.

Currently, *P. euphratica* fails to regenerate by seeds in the field, and root suckers are the main source of recruitment. Root suckers occur in most forest gaps. However, there is very low survivorship of root suckers, which leads to the failure of regeneration in many forest gaps. Although grazed root suckers may not initially die, sheep grazing has significant detrimental effects on their growth. Thus, sheep grazing delays the time taken for root suckers to develop into adult trees. The possibility of death for root suckers increases as the developing process increases in harsh environments.

In conclusion, *P. euphratica* has much potential for both sexual and asexual reproduction. There are several possible actions that may help to preserve and restore *P. euphratica*. Concrete canals in *P. euphratica* forests may limit opportunities for seedling establishment along banks. Additional work is needed to determine the effect of removing concrete canals on *P. euphratica* establishment. Altered stream flows may also help to produce wet conditions favoring seed germination and establishment. Coordinated efforts with those in charge of regulating stream flow may help to encourage establishment if sufficient water is released during peak seed rain.

## Materials and Methods

### Ethics approval

All necessary permits were obtained for the described field studies, which were carried out in the National Natural Reserve of *Populus euphratica* in Ejina County, Inner Mongolia, China. The Ejina National Natural Reserve of *Populus euphratica* issued the permission for all our field studies. *Populus euphratica* is a protected species in China. In field studies we tried our best not to damage seedlings or individuals. The soil was replaced after investigation of root suckers. When we investigated ages of individuals, the relation between age and diameter at breast height (DBH) was adopted rather than potentially harmful techniques such as stem cutting or increment coring.

### Study site

This study was conducted in the National Natural Reserve of *P. euhpratica*, in Ejina, Inner Mongolia Autonomous Region, China (41°30′∼42°07′N, 101°03′∼101°17′E, with an altitude of 900 to 1600m, Fig. 5). The climate of the study area is arid continental with a mean annual potential evaporation of approximately 3700 mm, and a mean annual precipitation of only 37.9 mm. The mean annual temperature is 8.2°C, with highest and lowest mean monthly air temperatures of 26.4°C (July) and −11.9°C (January) respectively. The vegetation is characterized by riparian *P. euphratica* forests.

The Heihe River, the second largest inland river in China, is the main contributor to the oasis, and groundwater in this region is mostly fed by the Heihe River [24], [26]. However, there has been a persistent decline of water supply as a result of extensive overuse of surface water for irrigation and for economic development in the upper reaches of the river since the 1950′s [27]. A series of severe environmental problems were triggered, such as the disappearance of the river and terminal lakes and a severe decline of the groundwater level [28], [29]. Several national projects have been initiated to re-establish the local ecosystems since 1990 [22]. Annual water discharge to the Ejina Oasis has been regulated at around 6×10^8^ m^3^ since the execution of the Heihe River Water Allocation Scheme in 2000 (http://www.yellowriver.gov.cn).

### Seed dispersal of *P. euphratica*

Three plots were established in homogeneous *P. euphratica* forests in the National Natural Reserve of *P. euphratica* in Ejina in 2006. Density, height and diameter at breast height (hereafter DBH) of seed-dispersing *P. euphratica* trees were examined (Table 5). Seed rain of *P. euphratica* was measured in an area of 40 m×40 m in plot 1. Twenty-five seed traps were deployed in this sampling plot. Our seed trap is a plastic bowl with a diameter of 10cm and a height of 7cm. The bowls were filled with water to prevent collected seeds from escaping. They were placed in 5 transects, with 5 bowls in each of them (5×5). There was an interval of 10 m between two adjacent bowls and between two adjacent transects. According to kind of microhabitat the seed traps were divided into three groups: on turf (n = 11), on bare ground (n = 7) and under shrub (n = 8). *Populus euphratica* seeds in all seed traps were collected and counted daily during the seed-rain season (early July to late August). In addition, starting date and duration of seed dispersal of each female adult *P. euphratica* individual were recorded in all three sampling plots (50 m×50 m) in 2008. Fruits of *P. euphratica* are capsules grouped in catkins. The capsules dehisce at maturity, and large amounts of cotton-containing seeds are released. Binoculars were used to observe seed dispersal of individuals. The earliest date open capsules were observed in any part(s) of the canopy was recorded as the starting date of seed dispersal of the individual. The date all capsules had opened and some of them had begun to fall was recorded as the ending date. Observations were carried out daily in all three plots for 59 d from 1 July to 28 August, 2008.

**Table 5:** Density, height and diameter at breast height of seed-dispersing *P. euphratica* trees in the three seed-dispersal study plots.

	Plot 1	Plot 2	Plot 3
Location	N41°58′6"	N42°0′31"	N42°0′35"
	E101°5′12"	E101°13′54"	E101°13′47"
Density (stems/100m^2^)	7.6	4.8	5.0
Mean height (m)	7.3±1.3	7.8±1.2	8.6±1.2
Mean DBH (cm)	32.3±2.5	27.0±1.5	42.2±2.6

Values for mean height and DBH in the table are means ± SE.

### Germinability of *P. euphratica*

Freshly-matured seeds were obtained from different parts of canopies in 40 trees of different ages in the *P. euphratica* forest at Erdaoqiao in July 2008. The seeds were mixed after harvest and incubated in 5-cm-diameter Petri dishes at alternating temperatures of 30 (day) and 20°C (night) which simulates conditions in the *P. euphratica* forest in July, in cool white fluorescent light (100μmol m^−2^ s^−1^, 400–700 nm) for 12 h and in darkness for 12 h each day. Three kinds of substrates were used: filter paper, soil collected from the *P. euphratica* forest and silt on local river banks, each with five replications. Soil and silt were used to represent different habitats of nonflooded forests and flooded river banks, and filter paper was used to test potential germinability of *P. euphratica* seeds. Fifty seeds were spread evenly in each Petri dish after the substrate was moistened with distilled water, and then the dishes were wrapped with clear plastic film to slow evaporation. Germination (emergence of radicle) was monitored every 12h. When no additional seeds had germinated for 5d, experiments were terminated, and final germination percentages were determined.

### Asexual regeneration of *P. euphratica* in the field

Four separate observations were conducted to analyze asexual regeneration of *P. euphratica* in the field. The first observation was aimed at gaining a general understanding of the establishment of root suckers of *P. euphratica* in the forests. Five line transects, each 5 km long, were established in the nature reserve of *P. euphratica* in 2008, and 10 forest gaps on each transect were investigated. In total, 50 forest gaps were surveyed. The number of living and dead root suckers of *P. euphratica* and the area of the forest gaps were recorded.

A second observation was conducted to determine factors influencing occurrence and growth of *P. euphratica* root suckers. Growth characteristics of *P. euphratica* root suckers including density, height and basal diameter of root suckers were recorded in 60 forest gaps. In addition, environmental conditions were also investigated, including species composition and coverage of ground cover flora, human disturbance (sheep grazing or not), number of *P. euphratica* adults around the forest gaps, distance from these adult trees to root suckers, and moisture and compactness of soil. If there were sheep excreta and evidence of sheep browsing on root suckers in a forest gap, it was recorded as sheep grazed. Intensity of sheep grazing was divided into four levels: 0, no sheep excreta or browsing; 1, <20% of root suckers browsed; 2, 20%∼50% root suckers browsed; and 3, >50% root suckers browsed. About 30 g of soil were collected around roots of root suckers, and the oven drying method was used to determine moisture content. Five replications were made in forest gaps with more than five root suckers. Soil samples were collected around all the root suckers if there were less than five root suckers in a forest gap. Degree of compactness of soil was obtained by using a portable soil compaction tester (SCM-6100, Spectrum Technologies, Inc., USA).

In the third observation, living and dead root suckers were counted in 20 forest gaps. Height and basal diameter of each living root sucker, depth and diameter of roots on which root suckers sprouted, and area of forest gaps were recorded. Density and mortality of root suckers were calculated as:

Density  = Number of living root suckers/Area of a forest gap

Mortality (%)  =  (Number of dead suckers/Number of dead and living suckers) ×100.

In the fourth observation, areas of 20 sites and density of *P. euphratica* on them where this species had successfully regenerated in recent decades were investigated; ages of 50 *P. euphratica* individuals were recorded on each site. Ages were calculated by employing the relationship between age and diameter at breast height (DBH). Xu [30] made a very intensive study of the relationship between age and DBH by measuring DBH of *P. euphratica* aged 5 to 137 y. On the basis of his study, a regression equation was obtained:

*a* =  4.086+0.395 × *d*+0.028 × *d* ^2^ (adjusted R^2^ =  0.999, P<0.0001), where *a* is age, and *d* is DBH.

### Effect of soil moisture on occurrence of root suckers

Due to rapid changes in environmental conditions in deserts, it is difficult to explore a relationship between occurrence of root suckers and environmental conditions in field investigations. An experiment was conducted in July 2010 to determine if there was any linkage between occurrence of root suckers and soil moisture. Five roots (d = 0.9±0.05 cm) of *P. euphratica* were excavated from 0–20 cm soil depth in the forest at Erdaoqiao, and each was cut into 5 sections 10 cm in length. Each root section was buried at a depth of 20 cm in a plastic pot (30 cm ×30 cm ×30 cm) filled with sandy soil collected from 0–20 cm soil depth in the habitat. Before use, the soil was mixed and dried at 105°C in an oven for 48h. Weights of pots (W_p_), soil in each pot (W_s_) and root sections (W_r_) were measured before burial of root sections. Weight of water (W_w_) was determined when the pots were first watered after burial of root sections. Soil moisture (SM) was calculated as:
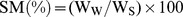



Five levels of soil moisture were used: 0 (CK), 5, 10, 15 and 20%. The experiment consisted of a completely randomized block design of five treatments, each with five replications. Each of the five sections from a root was haphazardly selected for incubation under one of the five soil moisture levels in pots in a greenhouse for 20 d. The pots were weighed daily, and water was added to make weights of the pots the same as they were first watered (W_p_+W_s_+W_r_+W_w_). The root sections were retrieved after 20 d and washed in running water. Number of buds of root suckers was counted.

### Statistical analysis

All data were analyzed using SPSS 13.0 (SPSS Inc. 2004). One-way ANOVA was used to estimate differences in seed rain density in different microhabitats, germination percentage among seeds incubated on different substrates, and number of buds on roots present in soil at different moisture contents. Multiple Comparisons procedure of least significant difference (LSD) was conducted when significant differences were found. In the second observation, ground cover plants were mainly *Sophora alopecuroides*, and few other species were found in *P. euphratica* forests. Thus density of *S. alopecuroides* was adopted to replace composition and coverage of ground cove plants when we analyzed correlation between growth characteristics of root suckers and influencing factors. Correlation between growth characteristics (density, height and basal diameter) of root suckers and influencing factors (density of *S. alopecuroides*, soil moisture at the depth of root suckers, degree of soil compactness, amount of adult individuals, mean distance from root suckers to adult individuals nearby, and sheep grazing) were analyzed using Bivariate Correlations procedure of SPSS. In the third observation, density, mortality, height and basal diameter of root suckers, depth and diameter of roots on which root suckers sprouted in the 20 forest gaps investigated were analyzed by using hierarchical cluster analysis (HCA). The cluster analysis was conducted using XLSTAT 2006 (Addinsoft, USA). The dendrogram was created using the Euclidean distance for dissimilarity scale for the Ward's method. The forest gaps were clustered into two groups by HCA, and differences in all the indices between the two groups of forest gaps described above were tested using Independent-Samples T Test procedure of SPSS. In the fourth observation, standard deviation (SD) and coefficient of variation (CV) were used to test variation in ages of individuals in a given site.
